# Perceptions of Successful Cues to Action and Opportunities to Augment Behavioral Triggers in Diabetes Self-Management: Qualitative Analysis of a Mobile Intervention for Low-Income Latinos With Diabetes

**DOI:** 10.2196/jmir.2881

**Published:** 2014-01-29

**Authors:** Elizabeth R Burner, Michael D Menchine, Katrina Kubicek, Marisela Robles, Sanjay Arora

**Affiliations:** ^1^Keck School of Medicine of the University of Southern CaliforniaDepartment of Emergency MedicineLos Angeles, CAUnited States; ^2^SC Clinical & Translational Science InstituteCommunity EngagementUniversity of Southern CaliforniaLos Angeles, CAUnited States; ^3^Children’s Hospital Los AngelesThe Saban Research InstituteLos Angeles, CAUnited States

**Keywords:** diabetes, Latinos, cellular phone

## Abstract

**Background:**

The increasing prevalence of diabetes and the associated cost of managing this complicated disease have a significant impact on public health outcomes and health expenditures, especially among resource-poor Latino patients. Mobile health (mHealth) may be the solution to reaching this group and improving their health.

**Objective:**

In this qualitative study, we examined nuances of motivation, intention, and triggers to action effected by TExT-MED (Trial to Examine Text Messaging for Emergency Department patient with Diabetes), an mHealth intervention tailored to low-income, urban Latinos with diabetes. TExT-MED is a fully-automated, text message-based program designed to increase knowledge, self-efficacy, and subsequent disease management and glycemic control.

**Methods:**

We conducted 5 focus group interviews with 24 people who participated in TExT-MED. We employed a modified grounded theory analytic approach—an iterative process of coding and immersion in the data used to recognize the patterns and links between concepts voiced by the participants. We coded data to identify themes of participant experiences, motivations, and responses to the program. We organized themes into a theory of TExT-MED’s action.

**Results:**

Participants enjoyed their experience with TExT-MED and believed it improved their diabetes management. Through analysis of the transcripts, we identified that the strengths of the program were messages that cued specific behaviors such as medication reminders and challenge messages. Our analysis also revealed that increasing personalization of message delivery and content could augment these cues.

**Conclusions:**

This in-depth qualitative analysis of TExT-MED shows that low-income Latino patients will accept text messages as a behavioral intervention. This mHealth intervention acts as a behavioral trigger rather than an education platform. Personalization is an opportunity to enhance these cues to action and further research should be conducted on the ideal forms of personalization.

## Introduction

The increasing prevalence of diabetes and the associated cost of managing this complicated disease have a significant impact on public health outcomes and health expenditures [1]. This is especially true among Latino patients, who have a higher rate of diabetes and diabetic complications than non-Hispanic White patients [2]. Additionally, the need for improved diabetes management is especially pronounced among resource-poor, low-income populations, as they fare worse than other patients [3]. Innovative and cost-effective solutions must be explored to improve health behaviors and health outcomes for these high-risk, low-income populations.

Mobile health (mHealth) may be the solution to reaching this group and improving their health. mHealth is the use of mobile devices to provide public health interventions and medical care. mHealth can consist of text messages, smartphone applications (apps), or Web-based interfaces and continues to evolve on other platforms. While mHealth has been shown to be effective in improving diabetes outcomes [4-6], it has not been widely tested in low-income, resource-poor populations, and is largely untested in Spanish-speaking populations. TExT-MED (Trial to Examine Text Messaging for Emergency Department patient with Diabetes) is the first program designed specifically for low-income Latinos. It involves is a six-month, fully-automated, text message-based program designed to increase knowledge, self-efficacy, and subsequent disease management and glycemic control [7]. The twice-daily text messages consisted of (1) educational/motivational messages, (2) medication reminders, (3) trivia questions, and (4) healthy living challenges. A randomized controlled trial of TExT-MED improved medication adherence compared to controls and was highly accepted by patients. Additionally, Spanish-speaking patients significantly improved their glycosylated hemoglobin (a measure of long-term blood glucose control) [7].

To better understand the findings of TExT-MED and to be able to develop and improve future interventions, we need to examine more than empirical data on outcomes. We must examine nuances of motivation, intention, and triggers to action. These are difficult to measure in novel interventions such as mHealth and among disadvantaged patients with poor health care access. Scales developed for patients from the mainstream culture may not correspond to the same concepts when delivered to low-income, low health-literacy, and non-English speaking populations [8]. Qualitative analytic techniques allow for a deeper understanding of the patient experience with mHealth, allowing us to maximize the benefits of this and future mHealth interventions. Qualitative analysis allows the participants to identify the components of the program they found most successful at achieving behavior change and improving their health rather than limit the responses to our preconceived ideas and hypotheses that are necessary for quantitative analysis. We conducted a series of focus groups and analyzed this data using a modified grounded theory approach to uncover those components of TExT-MED that participants perceived as most beneficial.

## Methods

In this qualitative study, we conducted a series of focus group interviews with people with diabetes who participated in TExT-MED. All 47 patients who had completed the earlier TExT-MED intervention and 6-month assessment were invited to participate in the focus groups through a series of phone calls and text messages. All 24 patients who agreed to participate were compensated for their time and travel. Focus groups were stratified by language and by gender to improve the comfort level of participants [9]. At the start of each focus group, an anchor survey was administered (Multimedia Appendix 1) and dinner provided to ease the start of the focus group [9]. Each session lasted 90 minutes to 2 hours, and a semi-structured question guide was used to prompt conversation (Multimedia Appendix 2). Experienced, bilingual team members facilitated the focus groups that were audio recorded. Focus groups were professionally transcribed verbatim and translated in the case of the Spanish language groups. These transcripts were uploaded to Dedoose, a Web-based qualitative analysis program [10]. This study was approved by the Institutional Review Board.

**Figure 1:**
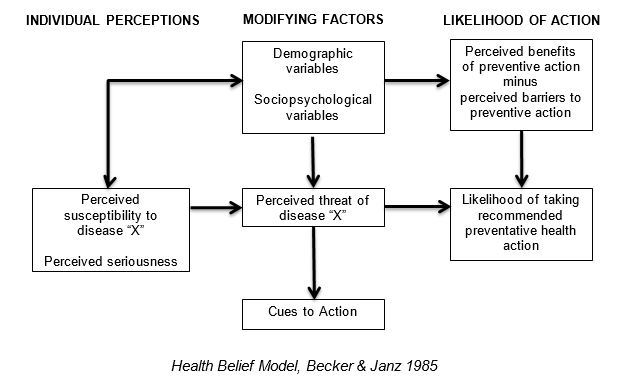
Health Belief Model.

We used a modified grounded theory analysis approach [11], an iterative process of coding and immersion in the data to recognize the patterns and links between the concepts voiced by the participants. Two project team members independently completed line-by-line open coding of two of the transcripts in order to maintain the detail of the data. An audit trail including memoing [11] was maintained to document all analytic decisions to increase trustworthiness (the qualitative analogue of validity). Through an iterative process of coding transcripts and discussing the coding schema, we developed a set of codes from the recurring ideas presented in the data, using the Health Belief Model as a sensitizing theoretical lens (Figure 1) [12]. After the development of the preliminary set of codes, we conducted multiple rounds of co-coding until consensus on code definitions was achieved. A test of coders’ agreement on application of these codes resulted in a Cohen’s pooled Kappa coefficient of greater than .7, showing good inter-coder reliability [13]. We then coded all transcripts through focused coding with the final codebook, while also checking to see if new codes needed to be created to capture new emerging themes. In order to better understand the relationships between codes, we then used a technique known as axial coding, comparing the situations where codes overlapped and the cases where these connections did not occur. This resulted in a grounded description of TExT-MED’s effect on participants. Saturation of codes was achieved by the fourth focus group. Our final codebook contained 27 codes and 24 sub-codes. We produced 327 pages of transcripts, which were read by three members of the research team (EB, MR, and KK.) In total, 759 individual excerpts were coded with between 1 and 11 codes each.

## Results

### Participant Summary

A total of 24 participants were successfully recruited and participated in a total of 5 focus groups (2 in English and 3 in Spanish; 2 for men and 3 for women). Study participants were between the ages of 26 and 76 years. They were 67% (16/24) female and 75% (18/24) Latino. A majority (58%, 14/24) spoke primarily Spanish. Almost all study participants (92%, 22/24) were uninsured. They had known of their diabetes diagnosis for between 1 to 45 years (Table 1). These characteristics did not differ significantly between the focus group participants and those patients that did participate in the focus groups (see Table 2).

Overall, the participants enjoyed the experience with TExT-MED and believed it had helped them improve their diabetes management and their “way of living”. Participants wanted the program and text-messages to continue and no one said TExT-MED had gone on too long. Many wanted the program to be expanded to more people and extended in length in future versions. Although not required, participants could add friends or family members to the program to receive messages. Many did, and the participants reported their friends and family found the program effective and enjoyable. Exemplary quotes included:

I am living well with this program. It has helped me enormously, enormously… I come because it’s worked for me. It has worked for me. A lot.

translated from Spanish

It has been a very good program for all the people with diabetes, and with this program we are better controlling our lives, our way of living.

translated from Spanish

I feel that it has helped me, it has helped me. And also the people that I signed up—I signed up five or six—it also helped them and they say, “When there is another program, invite us. It has worked.”

translated from Spanish

In general, participants were excited about this mHealth intervention as a novel way to learn about and manage their diabetes. In sum, the participants reported that TExT-MED helped them to take control of their diabetes and to make the behavior choices they believed would benefit their diabetes. Through the analysis, two specific strengths of the program and two opportunities for development emerged.

**Table 1:** Focus group participant characteristics (n=24).

Characteristic		Years or n (%)
Age		26-76 years (IQR^a^ 49-60 years)
**Gender**		
	Female	16 (67%)
**Ethnicity**		
	Latino	18 (75%)
	Non-Latino	6 (25%)
**Primary language**		
	Spanish	14 (58%)
	English	10 (42%)
**Insurance status**		
	Uninsured	22 (92%)
	Insured	2 (8%)
Number of years participants have known about diagnosis		1-45 years (IQR 6-15 years)

^a^IQR: interquartile range

**Table 2:** Focus group participant and non-participant characteristics.

Characteristic	Participants (n=24)	Non-participants (n=68)	*P* value
Age, mean (SD)	52.88 (10.25)	50.74 (10.42)	.387
Spanish language preference, n (%)	14 (58%)	50 (73%)	.171
Latino ethnicity, n (%)	18 (75%)	58 (85%)	.787
Female gender, n (%)	16 (62%)	46 (67%)	.667
Number of years participants have known about diagnosis, mean (SD)	11.6 (11.36)	9.8 (7.33)	.466

### Specific Strengths of Program: Medication Reminders

In every group, medication reminder messages were the most prominent and salient benefit mentioned. TExT-MED included various kinds of automated messages: educational/motivational messages, medication reminders, and healthy living challenges. Medication reminders consisted of specific prompts to take medication, as well as cues to have medications refilled or to take medications with them if they left the house. When discussing which messages most impacted their behavior, a participant stated:

Before you go to bed, it [the TExT-MED message] told me, like, “Don’t forget your medications and don’t eat nothing heavy before you go to bed”. And that was good.

And in this conversation between respondents:

That is what I like about the program, about the messages because they’re reminding me always to get our medicines.

Respondent 1

So that we won’t run out of medicines.

Respondent 2

Exactly. It helps us a lot because in reality, they’re good because they’re reminding us—

Respondent 1

They remind us not to forget to take the medicine and talk to the Doctor. Be on time with your medicines, do not wait to the last minute until we run out. We see all of this in those…in the messages.

Respondent 3; translated from Spanish

Participants found the specific behavior cued by the medication reminder messages easy to follow and to remember. They found both the direct cues to take medications, as well as the other tips to improve medication adherence, helpful and positive. While the participants generally were positive about the program, this specific component was the most frequently and emphatically mentioned as having the most influence on their diabetes management.

### Specific Strengths of Program: Healthy Living Challenges

Another major category of messages participants found impactful was the healthy living challenge. Participants received a message with a specific healthy behavior to perform (diet choice, physical activity, and mental health/relaxation) twice a week. There was no repeat reminder message to the challenge and participants did not have to respond that they performed the challenge. Participants mentioned these as particularly motivating. In regards to challenge messages, participants stated:

The challenges are good. The challenges you send us. One imagines that I see the message, and when I read the challenge, those are my challenges for the day. The challenges. It’s not every day but when they send challenges, they helped me a lot. I don’t answer them but I read them and I say, “I have to do this”. I motivate myself, like if I’m going to go walk. Or if I’m going to go eat a salad.

translated from Spanish

It’s like the little thing you put, you have in your head all the time, just thinking about it. It makes you think actually about what you’re doing to yourself actually. You know, if you’re not doing the proper thing. You know it’s like a head, a head reminder all the time. You wanna live longer, you know. It’s not just a little message, it’s what they, it’s what stays in your head about ‘em. And that’s the way I see it. More like a challenge.

Participants noted that these concrete behavior challenges were able to effect diet and exercise behavior and did not note this about educational messages. The participants perceived a need for a boost to live the healthier lives they already wanted. The healthy living challenges provided this boost. The emphasis on a specific behavior enabled participants to focus on one healthy change to make for that day. It seems that when this one goal was able to take root for that day, participants believed that it helped them in future diabetes management as well.

### Specific Development Opportunities: Increase Personalization of Message Delivery

In the design of TExT-MED, we opted to have all participants receive the same messages and to have automated message delivery. The messages did not include the patient name and the delivery schedule was not specified to anyone’s schedule. We did this to maximize the scalability of the program. However, participants specifically requested personalization of message delivery, including using their names and timing. Participants said they would have felt more cared for if the messages were more personalized. They also noted that medication reminders would come too early or too late to be most effective. They elaborated:

Can we ask for something more personal? Like “Hi, [states her own name],”…Computers can do so much now, nowadays; if you just said, “oh [own name]…just texting about your diabetes”…You see that it’s a text from you guys and say, “Hi, [own name], how are you doing today?”

I think that as technology advances, it will improve more-- in my case when I would receive messages about my pills, well I already have…I think that as technology improves, we can also see on the Internet, putting the hours at which we have to take our pills so that it can tell us exactly at what hours we need to do that. Because right now it is just a general announcement…But I think as time goes by and it becomes more personalized, it will help much, much more.

translated from Spanish

In summary, when asked about problems with the program, a lack of personalization was the most recurrent and prominent theme. Participants made the aforementioned suggestions about how their user experience could be improved. Additionally, they believed that this would make the program more effective. Specific development opportunities are to personalize messages with the patient name and to adjust the message delivery schedule according to patient preference and needs.

### Specific Development Opportunities: Increase Personalization of Message Content

Participants also wanted a program designed for their specific experiences with diabetes. They needed information that was relevant to the complications that they faced. Participants also noted that people at different stages of disease have different needs in establishing or changing disease management regimens:

Well, this is very personal, but the thing is we have to differentiate the levels of diabetes…For example in my case, when they took my, they cut my foot off. So then I had to look for, on my own, what types of exercises I have to do to help me to manage my diabetes and also learn how to live without my foot. So little things like that, like I say, everything is in general terms, because it’s a pilot program. But as it becomes more personalized, well maybe, we know that certain things affect this man and we can send him specific information for him.

translated from Spanish

The participants detailed several other areas that might vary between individuals including knowledge of food, exercise preferences, family, social support, and mental health needs. Tailoring an mHealth program to the specific needs of individuals would make it more impactful to their daily lives and health behaviors.

## Discussion

### Principal Findings

mHealth holds great promise for reaching resource-poor populations, especially low-income, urban Latinos. Text-messaging is a low-cost and feasible way to reach this high-risk group. More than three-quarters of Latinos in the United States own a cellular phone and, of these cell phone owners, 72% already use them to send or receive text messages [14]. Additionally, the potential for scalability of mHealth offers an opportunity to reach these patients cost-effectively. mHealth interventions have previously been shown effective as a tool in chronic diabetes management [4-6,15,16]. Currently studied mHealth programs for diabetes range from the highly intensive two-way and personalized messages [5,15-18] to one-way broadcast messages with no personalization at all [7,19,20]. While these reports are encouraging, neither the mechanism of mHealth action nor the optimal design of mHealth interventions is fully understood. A recent Cochrane review was conducted of text-messaging to improve self-care in patients with chronic disease [21]. This in-depth qualitative analysis of a low-cost, scalable mHealth program shows that patients will accept and enjoy uni-directional and broadcast messages. The nuances of motivation and behavior change that we uncovered using qualitative techniques gives us a greater understanding of how and why this mHealth intervention functioned. We identified specific cues to action as the most impactful messages and ways to make these cues more meaningful to patients. This allows us to create new programs that are more persuasive and effective.

Participants identified the most impactful and motivational messages to be the medication reminders and challenge messages—the two types of messages that cued concrete behavioral responses. According to the Health Belief Model applied to diabetes [12], individual patient factors affect the perceived threat of diabetes, the perceived benefits to acting to manage diabetes, and the barriers to taking these actions. Interventions can increase the perceived threat through cues to action, increase the perceived benefits of healthy behaviors, or decrease the perceived barriers to diabetes self-management. As behavior triggers were noted to be the most persuasive messages, this mHealth intervention appears to be most effective as a novel cue to action rather than educating patients to increase perceived benefits or decrease perceived barriers. Prior mHealth studies have shown success in affecting concrete behavior changes, suggesting mHealth takes on a different role than the traditional public health interventions that focus on educating patients to improve healthy behaviors [7,22,23]. Patients do not only need to be told why they should perform health behaviors, they need to be reminded, urged, and persuaded to undertake them. This is consistent with the model of persuasive design proposed by Fogg [24], that the likelihood of a behavior is a function of a person’s motivation and ability to perform that behavior, as well as the trigger to behavior provided by an intervention. The Fogg Behavior Model, while not specific to health interventions, helps to explain our findings. In this mHealth intervention, the behavior triggers were the most effective component.

Our analysis also uncovered ways to make these cues to action more meaningful; we found that patients desire some personalization of messages. This personalization may improve acceptability of an mHealth program for patients with diabetes when deployed in a larger population. Some of the personalization options identified by participants are feasible in an automated system, especially as the platforms for delivery of mHealth messages continue to advance. Using patient names and timing messages to the personal schedule of each patient would require a negligible increase in time in the initial set-up of messages for each patient, and could continue to run on an automated system. Tailoring messages to specific complications or years with diabetes may not be as easy to implement, but may make messages more persuasive and useful. However, this possible increase in effectiveness must be weighed against the cost of developing individualized programs for smaller and smaller subsets of patients. Further research should be conducted into how modules increasing a sense of personalization could be developed on a cost-effective basis and what types of personalization will result in more persuasive cues.

### Limitations

This qualitative analysis has several limitations. Participants were selected for their experience in the TExT-MED program and the groups were completed when all available participants had participated in a focus group rather than the common qualitative technique of terminating data collection when saturation of codes and concepts has been reached [11]. However, after the fourth group, no new codes were developed so we do not believe that we lost data through our sampling process. The structure of data collection (focus groups rather than individual interviews) may have limited the participants’ ability to express individual opinions. However, focus groups were selected over individual interviews due to the disadvantaged background of most of the participants, as Magill [25] asserts that focus groups allow the disenfranchised to voice disagreement with authority, which was critical to discovering areas of the program that required improvement. The perceptions and experiences of the respondents in this study came from a group of low-income, Latino adults who were largely uninsured. The nature of qualitative work limits the generalizability of findings to patients in different settings. Future researchers should consider focusing on younger populations as well as suburban and rural communities. Last, the use of a sensitizing concept in our modified grounded theory technique may have blinded us to other important themes. However, the use of the Health Belief Model as a theoretical lens was critical to organizing our understanding of the relationship between patient factors and the behavioral cues of TExT-MED. Data was carefully coded by multiple coders and memoing was employed to be certain that the themes developed came from the data and not from the sensitizing concepts.

### Conclusions

In spite of these limitations, this qualitative analysis has uncovered important information for further development of mHealth interventions for patients with chronic diseases, including diabetes. This analysis shows that a fully automated mHealth intervention tailored for low-income Latinos with diabetes is acceptable and persuasive for the target audience. This low-cost, scalable mHealth intervention acts as a behavioral trigger rather than a patient education platform. Increased personalization is an opportunity to enhance the strength of these cues to action. mHealth developers should focus on creating messages that are concrete behavioral triggers and personalizing the experience of users rather than in developing extensive educational messages. Future researchers should investigate whether increased personalization, especially in behavioral cues, results in improved health outcomes.

## Multimedia Appendix 1

Anchor survey used at beginning of focus groups.

## Multimedia Appendix 2

Interview guide used for focus group sessions.

## Abbreviations

TExT-MED: Trial to Examine Text Messaging for Emergency Department patient with Diabetes
