# Supplementary material for: Perceptions of Successful Cues to Action and Opportunities to Augment Behavioral Triggers in Diabetes Self-Management: Qualitative Analysis of a Mobile Intervention for Low-Income Latinos With Diabetes
Source: J Med Internet Res. 2014 Jan 29;16(1):e25. doi: 10.2196/jmir.2881 (PMC3936269; doi:10.2196/jmir.2881)
Supplement: Supplementary file 1 [file jmir_v16i1e25_app1.pdf]

Anchor Survey

Name: ____

Age: ____

Race: White Black Asian/Pacific Islander American Indian
Other Decline to State

Ethnicity: Latino Non-Latino Decline to State

Cellphone number: __________________________________

How long have you had diabetes? __________________

How long have you had a cell-phone? _______________

Do you have a regular telephone at home as well? ? Yes No

In your family, do people share a cellphone? Yes No Sometimes
How many people share the cellphone you use?_______________

Do you keep your cellphone with you all of the time? Yes No
Where else do you leave it?
- In a purse
- The car
- On a table,
- Other ________

How often do you receive text messages?
- Less than once a week
- Once a week
- Once a day
- Up to 5 times a day
- More than 5 times a day

How often do you send text messages to other people?
- Less than once a week
- Once a week
- Once a day
- Up to 5 times a day
- More than 5 times a day
