# Supplementary material for: Perceptions of Successful Cues to Action and Opportunities to Augment Behavioral Triggers in Diabetes Self-Management: Qualitative Analysis of a Mobile Intervention for Low-Income Latinos With Diabetes
Source: J Med Internet Res. 2014 Jan 29;16(1):e25. doi: 10.2196/jmir.2881 (PMC3936269; doi:10.2196/jmir.2881)
Supplement: Supplementary file 2 [file jmir_v16i1e25_app2.pdf]

Semi-structured Focus Group Guide

Introductory/Ice breaker questions: Name and….
-How many years have you had diabetes? How many years have you been coming to LAC+USC for care?
-What was your favorite part of participating in the program?

Main questions:
Thinking back to when you first found out you had diabetes, how did you take care of yourself?
Sub-prompts if not addressed: Who did you see to help you take care of it? (prompts if necessary: Medications, doctors, alternative medicine) What has been the hardest part? How do you learn about what you need to do to take care of yourself?

Do you take care of yourself differently now? (if necessary, prompt on diet, exercise, taking medicines)

Thinking back to when you first found out you had diabetes, and before the program, what kinds of things inspired you to take control of your diabetes?
Sub-prompts if not addressed: What kept you from managing it well?

What did you think would happen if you didn't manage your diabetes? Reword if necessary: What was the worst case scenario?

What kinds of things motivate you to change how you manage your diabetes?
Sub-prompts: Does it actually get you to change your behavior? What does?

Keep list of things to write up on board:
Thinking back to when you completed the TExT-MED program, how has all this changed? What do you think now? Try to touch on who they talk to about diabetes, how they learn new things about it, what they think their worse case scenarios is now, how they think they can manage it now compared to before.

Extra questions: if time
-How could TExT-MED be better?
-What types of messages made you think the most?
